# Risk of diabetes mellitus based on the interactive association between G6PD rs72554664 polymorphism and sex in Taiwan Biobank individuals

**DOI:** 10.1038/s41598-024-63361-9

**Published:** 2024-06-04

**Authors:** Yen-Lin Chang, Oswald Ndi Nfor, Ying-Hsiang Chou, Chih-Hsuan Hsiao, Ji-Han Zhong, Chien-Ning Huang, Yung-Po Liaw

**Affiliations:** 1 Department of Pharmacy, Taichung Veterans General Hospital, Taichung, Taiwan; 2 Department of Public Health and Institute of Public Health, Chung Shan Medical University, Taichung, 40201 Taiwan; 3 Center of Evidence-Based Medicine, Taichung Veterans General Hospital, Taichung, 407219 Taiwan; 4 Department of Radiation Oncology, Chung Shan Medical University Hospital, Taichung, 40201 Taiwan; 5 School of Medicine, Chung Shan Medical University, Taichung, 40201 Taiwan; 6 School of Medical Imaging and Radiological Sciences, Chung Shan Medical University, Taichung, 40201 Taiwan; 7 Department of Internal Medicine, Chung Shan Medical University Hospital, Taichung, 40201 Taiwan; 8 Institute of Medicine, Chung Shan Medical University, Taichung, 40201 Taiwan; 9 Department of Medical Imaging, Chung Shan Medical University Hospital, Taichung, 40201 Taiwan

**Keywords:** *G6PD* deficiency, Sex, Type 2 diabetes mellitus, rs72554664, Taiwan Biobank, Genetics, Biomarkers, Risk factors

## Abstract

The presence of *glucose-6-phosphate dehydrogenase* (*G6PD*) deficiency may increase the risk of type 2 diabetes mellitus (T2DM), with differing prevalence between males and females. Although *G6PD* deficiency is an X-linked genetic condition, its interaction with sex regarding T2DM risk among the Taiwanese population has not been fully explored. This study aimed to investigate the association between *G6PD* deficiency and T2DM risk in the Taiwanese population, focusing on the potential influence of sex. Data were obtained from the Taiwan Biobank (TWB) database, involving 85,334 participants aged 30 to 70 years. We used multiple logistic regression analysis to assess the interaction between *G6PD* rs72554664 and sex in relation to T2DM risk. The T2DM cohort comprised 55.35% females and 44.65% males (p < 0.001). The TC + TT genotype of rs72554664 was associated with an increased risk of T2DM, with an odds ratio (OR) of 1.95 (95% CI: 1.39–2.75), and males showed an OR of 1.31 (95% CI: 1.19–1.44). Notably, the G6PD rs72554664-T allelic variant in hemizygous males significantly elevated the T2DM risk (OR), 4.57; p < 0.001) compared to females with the CC genotype. Our findings suggest that the *G6PD* rs72554664 variant, in conjunction with sex, significantly affects T2DM risk, particularly increasing susceptibility in males. The association of the *G6PD* rs72554664-T allelic variant with a higher risk of T2DM highlights the importance of sex-specific mechanisms in the interplay between *G6PD* deficiency and T2DM.

## Introduction

*Glucose-6-phosphate dehydrogenase* (*G6PD*) deficiency is a prevalent genetic disorder affecting approximately 400 million people worldwide^1,2^. It is particularly common in the Mediterranean and Middle Eastern regions, with a higher incidence among males due to its X-linked inheritance pattern^3^. The *G6PD* enzyme plays a crucial role in protecting red blood cells from oxidative damage by producing nicotinamide adenine dinucleotide phosphate (NADPH), a reduced form of nicotinamide adenine dinucleotide^4,5^. Insufficient *G6PD* levels can lead to oxidative stress and compromise insulin secretion by beta cells^6^. Studies in animal models have demonstrated that *G6PD*-deficient mice have smaller pancreatic islets and impaired glucose tolerance, suggesting that beta-cell dysfunction and death may contribute to the progressive loss of beta cells observed in patients with diabetes^7^. Despite the clinical significance of this condition, research exploring the link between *G6PD* deficiency and diabetes is limited due to the scarcity of genetic data^6,8,9^.

*G6PD* deficiency arises from mutations in the *G6PD* gene. To date, over 200 *G6PD* mutations have been identified, including missense variants, multiple mutations, deletions, and intronic mutations^10,11^. The molecular epidemiological characteristics of *G6PD* deficiency have been extensively studied among Chinese populations, including the relationship between common genotypes and *G6PD* enzyme activity in homozygous males^12,13^. Notably, studies such as those by Fu et al.^14^ found associations between different gene mutations and varying levels of *G6PD* enzyme activity in Guangxi, China, while Chang et al.^13^ identified five mutations that account for more than 90% of *G6PD* deficiency cases in Taiwan. Among East Asian populations, the *G6PD* Kaiping variant (rs72554664, c. G1388A) is the second most common genetic variation, with a minor allele frequency (MAF) of 0.7%^15,16^. In China, the rs72554664 variant constitutes approximately 21.2 to 33.38% of all G6PD gene variations^17,18^. Individuals with *G6PD* Kaiping genotypes have been reported to exhibit a 60% reduction in *G6PD* enzyme activity^19^.

Sex is a non-modifiable risk factor for T2DM^20–23^, with significant differences in disease presentation, associated risk factors, and complications between males and females. More males than females are diagnosed with T2DM and fasting hyperglycemia. Males typically receive T2DM diagnoses at a younger age and with a lower BMI than females. Females with T2DM face a higher relative risk of cardiovascular disease and mortality compared to males. However, the potential interaction between G6PD genotypes and sex in relation to T2DM risk in East Asians remains unclear. Therefore, the current study aimed to investigate the interplay between sex and *G6PD* rs72554664 on T2DM risk, utilizing data from the Taiwan Biobank.

## Results

### Demographic characteristics of participants

The study included 85,334 participants, of whom 4235 (4.96%) had T2DM and 81,099 (95.04%) were healthy controls (Table 1). A significantly higher percentage of females (55.35%) had diabetes compared to males (44.65%). In the diabetic cohort, 15 males (0.35%) possessed the T allele, while 33 females (0.78%) had the TC genotype. The TT genotype was exceedingly rare, with its presence being nearly negligible in females and entirely absent in diabetic females. The average age was significantly higher in the diabetic group (58.41 years) compared to non-diabetic group (49.83 years). A higher percentage of diabetics were obese (36.25%) compared to non-diabetics (19.25%). Additionally, a greater proportion of individuals with T2DM reported drinking (6.04%) or smoking (10.91%) compared to those without T2DM (5.45% and 8.37%, respectively). Those with T2DM also had higher levels of HbA1c, AST, and ALT (p < 0.001).

**Table 1:** Demographic characteristics of study population.

	No type 2 diabetes (n = 81,099)	Type 2 diabetes (n = 4235)	p-value
Sex			< 0.001
Females	56,177 (69.27)	2344 (55.35)	
Males	24,922 (30.73)	1891 (44.65)	
Sex with genotype			< 0.001
Males with C	24,771 (30.54)	1876 (44.30)	
Males with T	151 (0.19)	15 (0.35)	
Females with CC	55,397 (68.31)	2311 (54.57)	
Females with TC	774 (0.95)	33 (0.78)	
Females with TT	6 (0.01)	0 (0.00)	
Age	49.83 ± 10.79	58.41 ± 7.83	< 0.001
Body mass index			< 0.001
Normal	41,203 (50.81)	1285 (30.34)	
Underweight	2874 (3.54)	46 (1.09)	
Overweight	21,412 (26.40)	1369 (32.33)	
Obese	15,610 (19.25)	1535 (36.25)	
Drinking			< 0.001
Never	74,816 (92.25)	3752 (88.60)	
Former	1860 (2.29)	227 (5.36)	
Current	4423 (5.45)	256 (6.04)	
Smoking			< 0.001
Never	66,738 (82.29)	3076 (72.63)	
Former	7576 (9.34)	697 (16.46)	
Current	6785 (8.37)	462 (10.91)	
HbA1c (%)	5.68 ± 0.61	7.48 ± 1.50	< 0.001
Bilirubin			0.279
Normal	75,439 (93.02)	3921 (92.59)	
Abnormal	5660 (6.98)	314 (7.41)	
AST (U/L)	24.89 ± 12.41	27.13 ± 13.71	< 0.001
ALT (U/L)	23.16 ± 20.27	28.63 ± 20.64	< 0.001
Drug allergy			< 0.001
No	73,407 (90.52)	3756 (88.69)	
Yes	7692 (9.48)	479 (11.31)	

*HbA1c* hemoglobin A1c, *AST* aspartate aminotransferase, *ALT* alanine aminotransferase.

### Risk factors for T2DM

Table 2 displays the factors assessed for T2DM. The TC + TT genotype was identified as a risk factor for T2DM compared to the CC genotype (OR = 1.95, 95% CI: 1.39–2.75). The risk was higher in males than in females (OR = 1.31, 95% CI: 1.19–1.44). Age, being overweight, obesity, HbA1c, former drinkers, and smokers were significantly associated with T2DM (ORs = 1.09, 1.26, 1.75, 3.60, 1.23, 1.19, respectively).

**Table 2:** Risk of type 2 diabetes among the study population.

	OR	95% CI	p-value
rs72554664 (ref: CC/C)			
TC + TT/T	1.95	1.39–2.75	< 0.001
Sex (ref: females)			
Males	1.31	1.19–1.44	< 0.001
Age	1.09	1.09–1.10	< 0.001
Body mass index (ref: normal)			
Underweight	0.70	0.49–1.00	0.050
Overweight	1.26	1.15–1.39	< 0.001
Obese	1.75	1.58–1.92	< 0.001
Drinking (ref: never)			
Former	1.23	1.02–1.48	0.028
Current	0.94	0.80–1.11	0.476
Smoking (ref: never)			
Former	1.19	1.06–1.35	0.004
Current	1.02	0.88–1.18	0.802
HbA1c (%)	3.60	3.47–3.73	< 0.001
Bilirubin (ref: normal)			
Abnormal	0.89	0.77–1.03	0.132
AST (U/L)	0.99	0.98–1.00	< 0.001
ALT (U/L)	1.00	1.00–1.01	0.086
Drug allergy (ref: no)			
Yes	1.30	1.15–1.46	< 0.001

*OR* odds ratio, *95% CI* 95% confidence interval, *HbA1c* hemoglobin A1c, *AST* aspartate aminotransferase, *ALT* alanine aminotransferase.

### Significant interaction between sex and rs72554664 genotypes on T2DM risk

An interaction was observed between sex and rs72554664 genotypes (p = 0.049). For the sex-specific genetic risk analysis (Table 3), male carriers of the T allele had higher odds of diabetes (OR = 3.44; 95% CI: 1.82–6.51) compared to C carriers. Disease risk was also higher among females with the TC + TT genotype compared to their CC genotype counterparts (OR = 1.66; 95% CI: 1.10–2.50). Age, HbA1c, and obesity remained significantly associated with T2DM in both males and females.

**Table 3:** Odds ratio for type 2 diabetes, with stratification by sex.

	Female				Male		
	OR	95% CI	p-value		OR	95% CI	p-value
rs72554664 (ref: CC)				rs72554664 (ref: C)			
TC + TT	1.66	1.10–2.50	0.016	T	3.44	1.82–6.51	< 0.001
Age	1.10	1.09–1.11	< 0.001	Age	1.08	1.07–1.09	< 0.001
BMI (ref: normal)				BMI (ref: normal)			
Underweight	0.85	0.57–1.25	0.413	Underweight	0.39	0.16–0.97	0.043
Overweight	1.42	1.26–1.60	< 0.001	Overweight	1.02	0.89–1.18	0.768
Obese	1.95	1.72–2.21	< 0.001	Obese	1.43	1.23–1.66	< 0.001
Drinking (ref: never)				Drinking (ref: never)			
Former	1.35	0.83–2.20	0.229	Former	1.24	1.02–1.51	0.032
Current	0.69	0.42–1.14	0.144	Current	0.99	0.83–1.18	0.904
Smoking (ref: never)				Smoking (ref: never)			
Former	1.07	0.74–1.55	0.705	Former	1.23	1.08–1.40	0.002
Current	1.14	0.80–1.63	0.480	Current	1.01	0.86–1.18	0.941
HbA1c (%)	3.80	3.62–4.00	< 0.001	HbA1c (%)	3.34	3.17–3.53	< 0.001
Bilirubin (ref: normal)				Bilirubin (ref: normal)			
Abnormal	1.11	0.88–1.40	0.385	Abnormal	0.79	0.66–0.95	0.011
AST (U/L)	0.99	0.98–1.00	0.007	AST (U/L)	0.99	0.98–1.00	0.029
ALT (U/L)	1.01	1.00–1.01	0.050	ALT (U/L)	1.00	1.00–1.01	0.772
Drug allergy (ref: no)				Drug allergy (ref: no)			
Yes	1.48	1.29–1.71	< 0.001	Yes	0.99	0.81–1.22	0.954

*OR* odds ratio, *95% CI* 95% confidence interval, *HbA1c* hemoglobin A1c, *AST* aspartate aminotransferase, *ALT* alanine aminotransferase, *BMI* body mass index.

### Stratification analysis by sex and rs72554664 genotypes on T2DM risk

Following the sex and genotype combinations (Table 4), males with the T allele had the highest odds for diabetes (OR = 4.57; 95% CI: 2.38–8.75) compared to the baseline group of females with the CC genotype. This was followed by females with the TC/TT genotype (OR = 1.63; 95% CI: 1.09–2.44) and males with the C allele (OR = 1.30; 95% CI: 1.19–1.43).

**Table 4:** Odds ratios for type 2 diabetes based on genotype combinations and sex.

	OR	95% CI	p-value
Sex and rs72554664 (ref: female with CC)			
Male with C	1.30	1.19–1.43	< 0.001
Female with TC/TT	1.63	1.09–2.44	0.018
Male with T	4.57	2.38–8.75	< 0.001
Age	1.09	1.09–1.10	< 0.001

*OR* odds ratio, *95% CI* 95% confidence interval, *HbA1c* hemoglobin A1c, *AST* aspartate aminotransferase, *ALT* alanine aminotransferase.

Adjusted for BMI, alcohol drinking, smoking, HbA1c, bilirubin, AST, ALT, and drug allergy.

## Discussion

Our study identified a significant elevation in the risk of type 2 diabetes mellitus (T2DM) among individuals carrying the *G6PD* rs72554664 variant, particularly the Kaiping variant denoted by the T allele, especially in hemizygous males. A notable interaction between genotype and sex was also observed. While previous research has explored the association between *G6PD* variants and diverse health outcomes, ours is pioneering in establishing a direct link between the *G6PD* Kaiping variant and T2DM, also being the first to assess T2DM risk considering both sex and the G6PD rs72554664 variant.

Recent literature suggests a link between *G6PD* deficiency and an increased incidence of diabetes^9,24–26^. The studies by Adinortey et al.^24^ and Santana et al.^26^ indicate a heightened risk of T2DM in individuals with *G6PD* deficiency, though, with varying ORs of 1.61 and 6.8, respectively. These disparities highlight the complex nature of *G6PD*’s role in T2DM development, potentially through oxidative stress or shared genetic predispositions^27^.

Our study focused on the *G6PD* genotype, which is associated with a reduction of around 60% in *G6PD* enzyme activity^19^ and found that the *G6PD* rs72554664-T allele significantly associates with increased T2DM risk across sexes, yet with a pronounced effect in males. This aligns with the previous findings cited above. This sex-specific disparity might be attributed to differences in sex hormones, with elevated testosterone levels in males linked to increased insulin resistance and T2DM risk^28,29^, underscoring the necessity for further investigation into these mechanisms.

The present study provides a new understanding of how the *G6PD* rs72554664 variant and gender jointly influence the risk of T2DM. It proposes two proactive measures: screening for the *G6PD* rs72554664 mutation in those with *G6PD* deficiency to evaluate their T2DM risk, and regular glucose monitoring for hemizygous males with the *G6PD* rs72554664-T risk allele. Such preemptive measures, alongside lifestyle modifications, could significantly mitigate the disease risk.

Our research boasts several strengths, including its basis in a population study that looked into how the *G6PD* rs72554664 variant and gender affect T2DM risk, with a sample size large enough to support comprehensive statistical analyses. Furthermore, the use of the Axiom Genome-Wide TWB Array Plate, a highly reliable and quality-controlled chip, enhances its relevance for genotyping individuals of East Asian descent, potentially improving clinical applicability.

However, the study has some limitations. Firstly, the sample size was restricted to East Asians aged 30 to 70 years, limiting the generalizability of the results to other ethnicities and age groups. Secondly, most participants in the Taiwan Biobank appeared healthy, and this could also limit applicability to hospital settings. Thirdly, the reliance on *G6PD* enzyme activity measurements for diagnosing *G6PD* deficiency might have overlooked individuals with mild or no symptoms, thereby complicating accurate clinical classification based on the disease’s presence. This highlights the importance of genetic screening for early risk detection and intervention even in the absence of characteristic symptoms. Fourthly, the underrepresentation of females with the TT variant may have precluded the statistical analysis, potentially resulting in an overestimation of disease risk in females with the TC and an underestimation in those with the TT genotype. Future research endeavors will delve into more nuanced subgroup analyses within the female population, aiming to delineate the genetic underpinnings of diabetes risk more accurately.

Our study, which incorporates genetic factors, offers valuable insights into the potential causal relationship between *G6PD* deficiency and T2DM. However, the mechanisms underlying this association require further investigation. Despite its suggestive findings, the observational nature of this study means it cannot conclusively establish causality. Therefore, additional well-designed prospective studies and randomized controlled trials are necessary to establish causality and identify potential interventions that could reduce the risk of T2DM in individuals with *G6PD* deficiency.

## Conclusions

In conclusion, the *G6PD* rs72554664 variant significantly influences T2DM risk among Taiwanese adults, with a heightened susceptibility in males. This reinforces the importance of genetic factors in understanding T2DM risk and underscores the need for further research to uncover the mechanisms behind this association and identify effective preventive strategies.

## Materials and methods

### Study participants

The cohort for this investigation comprised Taiwanese citizens who voluntarily participated in the Taiwan Biobank program from 2008 to 2019. Comprehensive data on demographics, lifestyle, anthropometry, and genetics were obtained through questionnaires, physical assessments, and biochemical assays at medical recruitment centers. Written informed consent was obtained from all participants before any assessments were conducted at various centers. The initial pool of participants included 88,347 individuals, and 85,334 individuals (4235 T2DM patients and 81,099 controls) were included in the final analysis after excluding those with incomplete questionnaire responses (n = 3013). The Institutional Review Board of Chung Shan Medical University approved this study (CS2-21203, CS1-20009).

### Whole-genome genotyping

The National Center for Genome Medicine (Academia Sinica, Taiwan) was responsible for the genomic data. The single nucleotide polymorphism (SNP) was identified using the Axiom Genome-Wide Array Plate (Affymetrix, Santa Clara, CA, USA). Following a thorough literature review, the *G6PD* rs72554664 variant was chosen, where the T allele was the minor allele. Females might exhibit a homozygous deficient state, indicated by possessing two *G6PD* deficient alleles (TT), or they could be heterozygous, carrying one G6PD deficient allele and one normal allele (TC). Individuals may also demonstrate a homozygous normal state, having two *G6PD* normal alleles (CC). Conversely, males, with only one allele, can be categorized as hemizygous deficient (T) or hemizygous normal (C) concerning *G6PD* deficiency. To assess genetic associations, the dominant model was utilized. For the quality control, the SNP had a call rate  >0.95 while the minor allele frequency (MAF) was 0.01. The p-value for the Hardy–Weinberg Equilibrium (HWE) was 0.07.

### Definition of diabetes and other variables

The primary objective of this study was to investigate the occurrence of T2DM, defined as having a fasting glucose level of ≥ 126 mg/dl, a glycosylated hemoglobin A1c value of at least 6.5%, or self-reported diabetes. The BMI was calculated as weight divided by height squared (kg/m^2^), and the following categories were utilized: 18.5 ≤ BMI < 24 for normal weight, 0 ≤ BMI < 18.5 for underweight, 24 ≤ BMI < 27 for overweight, and BMI ≥ 27 for obesity. Information on lifestyle characteristics such as alcohol consumption and smoking habits were obtained through a face-to-face interview with a staff from the TWB. Participants who consumed more than 150 mL/week of alcohol for at least 6 months were designated as current drinkers, while those who had stopped drinking for at least 6 months were categorized as former drinkers. Nondrinkers were those who had no history of drinking. Current smokers were defined as those who had smoked continuously for at least 6 months, while former smokers were individuals who had stopped smoking for at least 6 months. Nonsmokers were those who had no history of smoking. Hemoglobin A1c (HbA1c), aspartate aminotransferase (AST), and alanine aminotransferase (ALT) levels were obtained from the TWB database. Bilirubin levels were measured in milligrams per deciliter (mg/dL). For participants aged 30 to 60, bilirubin levels between 0.3 and 1.2 mg/dL were considered normal. For those over 60, bilirubin levels from 0.2 to 1.1 mg/dL were classified as normal. Levels outside these ranges were classified as abnormal. Self-reported medication allergies were also collected from the TWB questionnaire.

### Statistical analyses

For data management, SAS version 9.4 (SAS Institute, Cary, NC, USA) and PLINK 1.90 beta were employed. Continuous and discrete variable distributions were assessed using *t*- and Chi-square tests, respectively, to compare males and females. Logistic regression was utilized to examine the association of T2DM with rs72554664 and sex, as well as the interaction between sex and rs72554664. All models were adjusted for potential confounders to ensure the robustness and validity of the findings. The confounders included age, body mass index, alcohol drinking, cigarette smoking, hemoglobin A1c, bilirubin, aspartate aminotransferase, alanine aminotransferase, and drug allergies. The statistical significance threshold was set at p-value < 0.05.

### Ethics statement

The study protocol followed the guidelines for human studies and was conducted ethically in accordance with the World Medical Association Declaration of Helsinki. The study protocol was reviewed and approved by the Institutional Review Board of Chung Shan Medical University approved this study (CS2-21203, CS1-20009).

## Data Availability

The study data are available upon request from the corresponding author.
